# Early origins of asthma (and allergy)

**DOI:** 10.1186/s40348-016-0056-4

**Published:** 2016-08-10

**Authors:** Michael Kabesch

**Affiliations:** Department of Pediatric Pneumology and Allergy, University Children’s Hospital Regensburg (KUNO), Campus St. Hedwig, Steinmetzstr. 1-3, 93049 Regensburg, Germany

**Keywords:** Asthma, Wheezing, Epigenetics, Interaction, Gene by environment

## Abstract

Asthma is the most common chronic disease starting in childhood and persisting into adulthood in many cases. During childhood, different forms of asthma and wheezing disorders exist that can be discriminated by the mechanisms they are caused by. Specific genetic constellations and exposure against environmental factors during early childhood and in utero play a decisive role in the early development of the disease. Epigenetic mechanisms which are master regulators of gene transcription and thus govern the accessibility and use of genome information, have recently been identified as a “third power” determining many features in the early development of asthma and allergy.

Asthma is the most common chronic disease starting during childhood and more than 300 million individuals are affected across the world according to the world health organization (WHO). Genetic predisposition and environmental risk factors determine the development of asthma during childhood. However, recent analyses suggest that asthma is not one disease but a syndrome of different phenotypes and endotypes that can be discriminated by their clinical manifestation, their course, and their underlying mechanisms. It is a peculiarity of asthma that its diagnosis is difficult to secure in the absence of a diagnostic gold standard. This is especially true in early childhood, when many children—and estimates go up to 50 %—experience wheezing in the course of a common infection of the upper airways at least once [1]. Recurrent wheezing due to airway obstruction is the leading symptom of asthma accompanied by coughing with and without mucus production. However, these are also common features observed in common colds during early childhood. In children with asthma, wheezing during upper airway infection is common during early childhood. Therefore, in most asthma patients, the definite onset of disease is only recognized retrospectively, once the diagnosis is made clear in preschool age while early childhood remains a dark spot in asthma diagnosis as a strong overlap with other forms of infection associated wheeze exist.

Many classifications for asthma and wheeze have been suggested such as the clinical discrimination of different asthma forms based on allergy and severity of respiratory symptoms. While this seems to be useful and stable over time in adults with asthma [2], clinical classifications of asthma and wheeze seem to be much less reliable in childhood. Using the clinical phenotypes multitrigger wheeze, unremitting wheeze, recurrent unremitting wheeze, frequent wheeze, episodic wheeze, and a doctor’s diagnosis of asthma [3], one study recently investigated how these phenotypes compare to a latent class analysis of the data in the course of asthma during early childhood in a birth cohort (Fig. 1). Latent class analysis is a hypothesis free self-organization of data according to clusters, and as shown in Fig. 1, this latent class analysis discriminates very well between the different courses of wheezing diseases in early childhood while clinical classification does not. That this is a valid observation becomes clear, as very similar phenotypes of early childhood wheeze have been defined by latent class analyses in different populations [4].

**Fig. 1:**
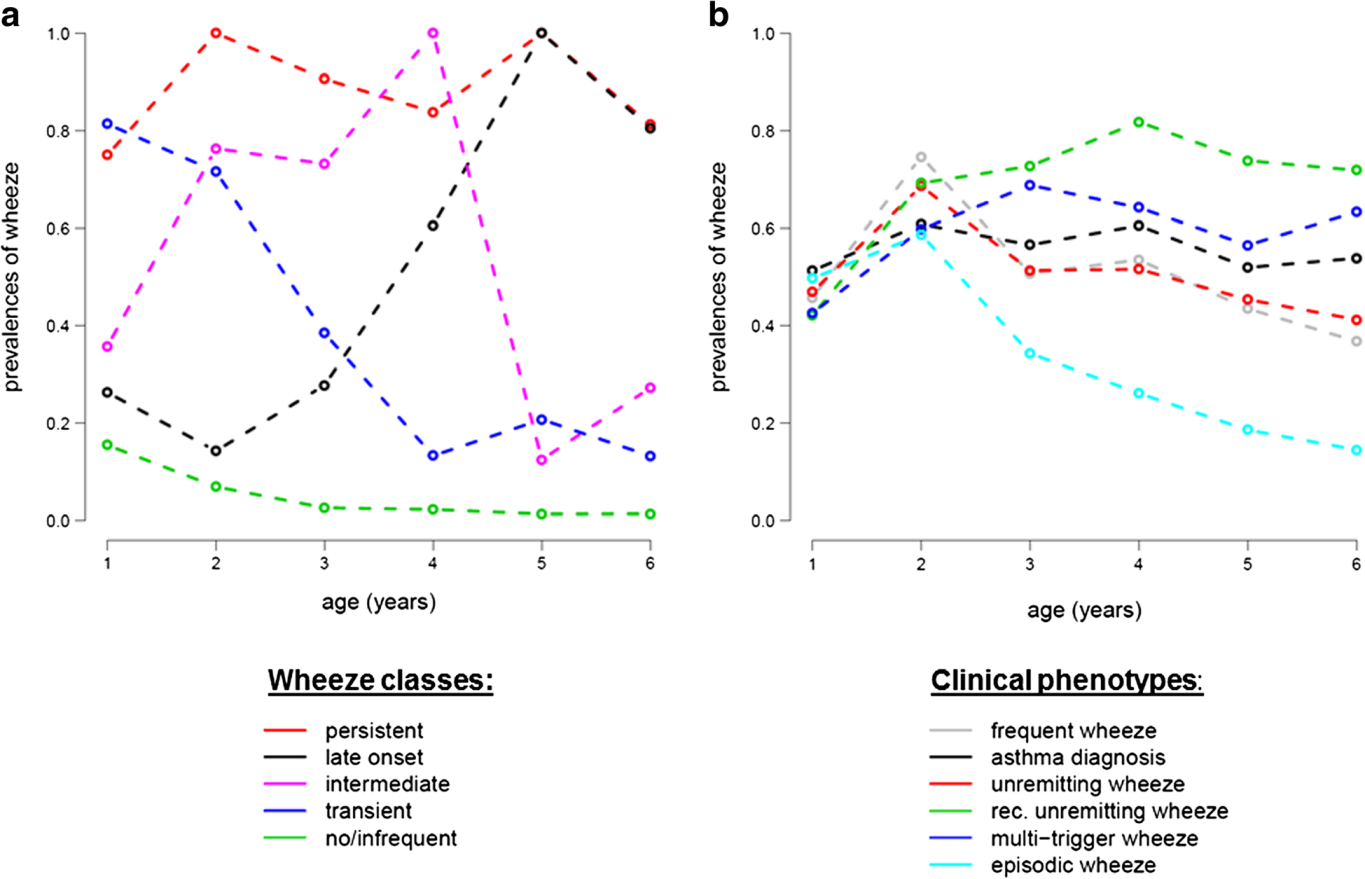
Course of wheeze prevalences in latent class analysis (LCA) and clinical phenotypes. **a** Prevalences of current wheeze (i.e., in the last 12 months) in the first 6 years of life are shown for the five class solution of the LCA. **b** Wheeze prevalences in the first 6 years of life stratified for clinical phenotypes as defined in the text and reference [2], used with permission

What becomes clear from these data is that most individuals with asthma develop their symptoms very early and that different phenotypes of wheezing exist already from very early on. Therefore, mechanisms involved in the development of asthma must be present very early. Very early environmental factors, potentially even fetal exposures and inborn genetic factors, are therefore a natural candidate to investigate for their impact on asthma.

Indeed, association between genetic mutations and asthma has been described [5]. Interestingly, the genetic impact is much stronger on asthma starting in childhood than on asthma starting later in life [6]. Different genes are involved in asthma and potentially, different endotypes of asthma are defined by their underlying genetic background. The strongest genetic signal for childhood asthma is an association from chromosome 17q21 where the genes *ORMDL3* and *GSDMA* are localized. For *ORMDL3*, a potential role in asthma by influencing endoplasmatic reticulum stress reaction, oxidative stress reaction, calcium homeostasis, and phospholipid metabolism has been shown [7–10]. Interestingly, genetic susceptibility for asthma is different from susceptibility for allergic sensitization, atopic dermatitis, and other allergic phenotypes suggesting that disease-specific factors are present rather than one general genetic susceptibility for all atopic diseases. The individual asthma and wheezing forms as defined by clinical classification or latent class analysis also have a strong genetic component (except for transient wheezing). In fact, family history of asthma is the strongest risk factor for most of these phenotypes. When analyzing the impact of *ORMDL3*-related mutations on these phenotypes in detail, a strong allele specific increase in the risk for persistent wheeze (3-fold) and a diagnosis of asthma (2-fold) was observed [3]. In terms of environmental factors, allergen exposure showed the strongest association with wheeze phenotypes in that analysis, specifically with late onset wheeze.

It is important to note here that the current concept in asthma and allergy is not that either environment or genetic factors cause the disease but that these two factors do interact in the development of the disease. It has also been realized that age and timing play an important role in these gene-by-environment interactions. In general, it is thought that the younger an individual is, the more susceptible it is to environmental effects, may they be protective (such as early exposure to farm life) or harmful (such as in utero exposure to maternal smoking). If the genetic setup would be stable over a lifetime, these differences in effect weight would not be the case, and thus, a further player must be involved to explain the increased impact of these early gene-by-environment interactions on disease development. A factor that may explain these timing effects may be epigenetics.

Epigenetics defines heritable mechanisms that influence gene function through gene expression not caused by changes in the nucleotide sequence of the genetic code itself. Epigenetic mechanisms in humans are post-transcriptional histone modification, small and other non-coding RNAs (siRNA and miRNAs) and DNA methylation as depicted in Fig. 2 [11]. Chromatin configuration, that is the meta-organization of the genome, controls accessibility of DNA for transcription and thus the use of DNA-based information. Histone proteins interact with DNA to either lead to open, accessible chromatin (euchromatin) or closed, non-accessible chromatin (heterochromatin). Histones and DNA form the nucleosome, which is the basic unit in which the genome is organized. The nucleosome consists of an octamer of histone proteins around which exactly 146 base pairs of DNA are wrapped. Histone proteins can be chemically modified by acetylation, methylation, phosphorylation, and ubiquitylation [12], changing their configuration and in turn the shape of the nucleosome from closed (e.g., acetylated) to open (e.g., deacetylated). If chromatin remains closed in the area where a certain gene lies, that gene cannot be transcribed and not translated into protein. Enzymes such as histone acetylases and histone deacetylases regulate histone modification by adding or retracting acetyl groups and thereby closing or opening accessibility of the nucleosome.

**Fig. 2:**
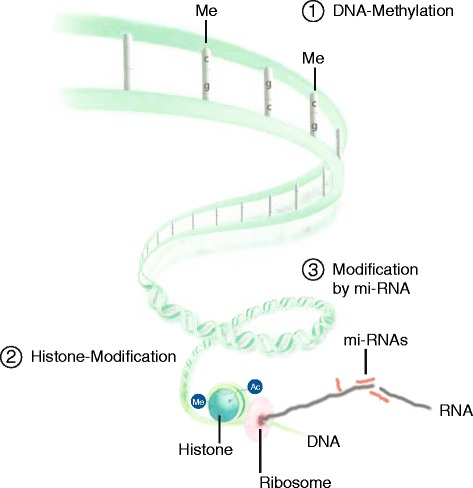
Epigenetic mechanisms in humans. The genome has a three-dimensional structure. The DNA is configured as an alpha helix of nucleotides. (*1*) The addition of methyl groups to cytosines changes the configuration of the helix and hinders the transcription machinery to attach to DNA. The DNA is further organized in nucleosomes which contain 146 basepairs of DNA and eight histone proteins (4 × 2 histone proteins). (*2*) Modification of histones by methylation or acetylation changes the accessibility of DNA at the site of these nucleosomes. Once mRNA is produced, (*3*) interfering mi-RNA can regulate transcription efficiency before translation into protein can occur (adapted from figure 7.6, chapter on genetics by Michael Kabesch in Pädiatrische Pneumologie, Editors Erika von Mutius et al., Springer, ISBN 978-3-642-34827-3)

The second important epigenetic mechanism controlling gene expression by regulation of transcription accessibility is the methylation of DNA [13]. Methylation is the modification of the pyrimidine ring of the DNA nucleotide cytosine by a methyl group. Cytosines are randomly spread throughout the genome but they are also found in a specific CpG configuration (which is a dinucleotide formed by cytosine and guanine) in regulatory regions of the genome such as gene promoters. Normally, the majority of CpGs are methylated acting as “stop signs” for transcription, and thus, areas where CpGs occur methylated are closed for transcription. Induction of transcription is an active and targeted process that needs demethylation of a specific locus by enzymes.

Once transcription from DNA to mRNA has occurred in open, unmethylated euchromatin, one further and last epigenetic mechanism controlling gene expression is RNA interference. MicroRNAs (miRNAs), which are small single-stranded non-coding RNAs highly conserved across species, act as regulators of transcription efficiency by degrading and thus counteracting mRNA [14]. Sequences for miRNAs are localized in intergenetic and intronic regions (between genes and between exons of genes) and expressed in a tissue-specific manner.

All three mechanisms described above are capable of regulating biologically relevant and disease-associated processes. They are responsive for environmental influences and allow for rapid adaption of genome use to changing environments and reaction to danger signals in a cell and tissue-specific, well-balanced manner.

Epigenetic mechanisms may be involved in the induction of asthma due to environmental tobacco exposure (ETS) or even worse, tobacco smoke exposure in utero. ETS is the single most important environmental factor increasing asthma risk by approximately 20 % [15]. Unfortunately, millions of children are exposed to tobacco smoke from very early on and within families, and this pattern of exposure is present over generations. What this can induce was very impressively shown recently in a rat animal model [16], when pregnant mother animals (F0 generation) were exposed to tobacco smoke. Their offspring (F1 generation) showed massively impaired lung function from early on and until adulthood, even though this F1 generation was never exposed to tobacco smoke. Even more astonishing, the next generation (F2 generation) showed lung function alterations, although it was only their grandparent generation that was once exposed to tobacco smoke. Lung function impairment was accompanied by epigenetic changes (a) in the lung and (b) in the germ line persisting over generations. In the lungs, differences in histone (H3 and H4) acetylation were found across generation and methylation signatures were changed in testis and ovaries. In humans, this smoking grandparent effect on asthma development in the offspring was also reported [17] but no epigenetic analyses were available in that study at the time. However, epigenetic effects of tobacco smoking are very well documented [18] and even after in utero tobacco exposure, massive changes in methylation signatures were found in newborns. However, for these children, data on disease development are not (yet) available [19].

High throughput assessment of epigenetic signatures on a genome wide level is so far available for methylation analyses. In humans, multiple methylation signatures across the genome are significantly associated with total IgE levels across populations as recently reported [20]. These differences in methylation marks are found in genes already previously linked to allergies and IgE levels such as *IL4* and *IL5 receptor* but also novel genes such as *LPCAT2* (involved in lysophospholipid metabolism), *L2HGDH* (a mitochondrial oxidoreductase), and in the transcription factor *ZNF22*. In this study, patients with established allergies and asthma were investigated at one time point only. Thus, methylation differences may in part be due to active asthma and not the reason for asthma, as disease itself induces methylation changes, e.g., when immune genes are activated during the disease, demethylation of that locus has to occur.

In a recent hallmark study [21], the effect of maternal smoking during pregnancy on epigenetic signatures in mothers and newborns was assessed on the methylation and chromatin level using genome wide bisulfite and chromatin immune precipitation sequencing. Interestingly, differently methylated regions due to smoke exposure varied between mothers and children suggesting that in children other epigenetic mechanisms than in actively smoking adults are at work. Chromatin of children transformed into a hyper-activated and more accessible chromatin state in response to maternal smoking, especially in regulatory regions of the genome, so called enhancer regions. Repeated analysis 1 and 4 years after birth showed that tobacco smoke-induced epigenetic signatures were stable for up to 4 years after birth, suggesting a long-term impact of tobacco-related adverse health effects. Indeed, epigenetic patterns identified in the discovery cohort were validated in two large German cohorts of children, the LINA and LISA studies where specific epigenetic marks also associated with the occurrence of late onset wheeze.

To also assess the influence of environmental exposure and disease on epigenetic signatures over time, we analyzed methylation of asthma and allergy candidate genes at birth and at age 4.5 years in children growing up on farms (exposure) and children developing asthma (disease) in a crossover design [22]. We investigated whether epigenetic patterns in asthma candidate genes (a) are influenced by farm exposure in general, (b) whether these change over the first years of life, and (c) whether these changes may contribute to the development of asthma. In cord blood, some candidate regions were hypomethylated in DNA from farmers’ as compared to nonfarmers’ children, while others were hypermethylated. Changes in methylation over time occurred in 15 gene regions. Interestingly, these differences clustered in genes highly associated with asthma (*ORMDL* family) and IgE regulation (*RAD50*, *IL13*, and *IL4*), but not in T-regulatory genes (*FOXP3*, *RUNX3*). Thus, it seems that DNA methylation patterns change significantly in early childhood in specific asthma- and allergy-related genes in peripheral blood cells, and early exposure to farm environment may influence methylation patterns in distinct genes.

When we analyzed children growing up on farms for epigenetic signatures across the genome in a pilot project, we found that methylation marks differed strongest between farm children and non-farm children at birth (measured in cord blood), while differences between children with and without asthma were more pronounced at age 4.5 compared to at birth. However, the most significant differences were observed when changes in methylation over time from birth to age 4.5 years were compared between children that developed asthma and those that did not (own unpublished data). When pathway analyses were performed, methylation differences clustered in signaling cascades of the innate immune system.

Modification of epigenetic signatures is a potential therapeutic concept. In fact, corticosteroids, the most potent medication for asthma, is a strong epigenetic modifier by acting upon histone acetylation. However, corticosteroids are untargeted epigenetic modifiers and targeted manipulation of epigenetic signatures in a cell-specific and timely manner is what one would like to achieve. A better understanding of the natural mechanisms in epigenetics involved in asthma and allergy will be the first step necessary towards targeted epigenetic therapy and early prevention. Pharmaceutical industry has already invested heavily in the development of such drugs [23] for their use in cancer and there is hope that learning from these experiences, epigenetic therapy will also reach asthma.

Taken together, current evidence indicates that asthma has early origins and differences in preschool forms of asthma and wheezing exist. Genetic and early environmental factors such as in utero tobacco smoke exposure influence disease development. These early environmental factors imprint on epigenetic signatures that can be measured today. Epigenetic signatures change over time and (a) may allow to identify children susceptible for the development of asthma before clinical disease is present, (b) are associated with the presence of asthma, and (c) may be changed by therapy.
